# When Infections Are Found: A Qualitative Study Characterizing Best Management Practices for Central Line-Associated Bloodstream Infection and Catheter-Associated Urinary Tract Infection Performance Monitoring and Feedback

**DOI:** 10.3390/nursrep14020080

**Published:** 2024-04-27

**Authors:** Alice A. Gaughan, Sarah R. MacEwan, Megan E. Gregory, Jennifer L. Eramo, Laura J. Rush, Courtney L. Hebert, Ann Scheck McAlearney

**Affiliations:** 1 The Center for the Advancement of Team Science, Analytics, and Systems Thinking in Health Services and Implementation Science Research (CATALYST), College of Medicine, The Ohio State University, Columbus, OH 43202, USA; Alice.Gaughan@osumc.edu (A.A.G.); Sarah.MacEwan@osumc.edu (S.R.M.); Megan.Gregory@ufl.edu (M.E.G.); Jennifer.Eramo@osumc.edu (J.L.E.); Laura.Rush@osumc.edu (L.J.R.); Courtney.Hebert@osumc.edu (C.L.H.); 2 Division of General Internal Medicine, College of Medicine, The Ohio State University, Columbus, OH 43202, USA; 3 Department of Biomedical Informatics, College of Medicine, The Ohio State University, Columbus, OH 43210, USA; 4 Department of Health Outcomes & Biomedical Informatics, College of Medicine, University of Florida, Gainesville, FL 32608, USA; 5 Department of Family and Community Medicine, College of Medicine, The Ohio State University, Columbus, OH 43201, USA

**Keywords:** infection prevention, healthcare-associated infections, best practices, management practices, qualitative methods, HAI performance monitoring and feedback

## Abstract

Healthcare-associated infections (HAIs) remain a significant patient safety problem that can lead to illness and death, despite the implementation of clinical bundles to prevent HAIs. Management practices can support HAI prevention, but their role in HAI performance monitoring and feedback is not well understood. To address this knowledge gap, we previously conducted semi-structured interviews with staff at 18 hospitals to examine the role of management practices around the prevention of central line-associated bloodstream infections (CLABSIs) and catheter-associated urinary tract infections (CAUTIs). Interview transcripts were analyzed to identify themes related to HAI performance monitoring and feedback. The current analysis focuses on 10 higher-performing hospitals that were successful in preventing CLABSIs and CAUTIs. These institutions had robust practices including timely event analysis, leadership engagement, and multidisciplinary participation in HAI reviews. Across these sites, we found common goals including investigating HAIs without blame and identifying opportunities for improvement. Management practices such as timely analysis of HAIs, collaboration between facility leadership and multidisciplinary team members, and a focus on identifying the failure of a procedure or protocol, rather than the failure of staff members, are all approaches that can support infection prevention efforts. These management practices may be especially important as hospitals attempt to address increases in CLABSI and CAUTI rates that may have occurred during the coronavirus pandemic.

## 1. Introduction

Healthcare-associated infections (HAIs), including central line-associated bloodstream infections (CLABSIs) and catheter-associated urinary tract infections (CAUTIs) contribute to increased patient morbidity and mortality while adding unnecessary costs to healthcare [1,2,3]. Since HAIs are largely preventable, clinical bundles have been created and promulgated to reduce the occurrence of HAIs. Clinical bundles for device-related HAIs like CLABSIs and CAUTIs include both tools and policies related to the insertion, maintenance, and removal of those devices [4,5]. Despite this clinical focus, success in HAI reduction has been variable, suggesting that other factors, such as management practices, may have a role in infection prevention [6]. Improving our understanding of the procedures and/or protocols implemented as part of the HAI performance monitoring and feedback process can inform management practices, identify opportunities to improve clinical practice, and prevent future infections.

The importance of investigating the cause of each HAI is supported by the principles and practices of a patient safety culture which highlights the value of opportunities to learn from failure, especially when the goal is to minimize high-risk patient safety errors [7]. While the value of investigating HAIs is evident, little is known about the details of how this is achieved in hospitals that are more successful at preventing HAIs. This study, therefore, examines various approaches to the CLABSI and CAUTI performance monitoring and feedback process to characterize those management practices that may improve patient safety outcomes related to these infections.

## 2. Materials and Methods

### 2.1. Study Design

We conducted site visits at hospitals in the United States (US) between September 2017 and November 2019 to study management practices around HAI prevention. As part of this work, we explored the HAI performance monitoring and feedback processes at these hospitals, with a primary focus on central line-associated bloodstream infections (CLABSIs) and catheter-associated urinary tract infections (CAUTIs). The Standards for Reporting Qualitative Research (SRQR) checklist was used when writing this report [8].

### 2.2. Study Sites and Participants

Hospital performance data, including infection rates for CLABSIs and CAUTIs, are collected by the Centers for Medicare & Medicaid Services and shared through Hospital Care Compare on Medicare.gov [9]. Using the publicly available Hospital Care Compare data focused on hospital performance for CLABSIs and CAUTIs, we aimed to recruit a collection of hospitals designated as better than the national benchmark; average or no different than national benchmark; or worse than the national benchmark.

Over 40 hospitals with different levels of success in preventing CLABSIs and CAUTIs were invited to participate in the study by emailing information to chief hospital executives at each site. Using a purposive sampling approach, we aimed to recruit hospitals with a variety of organizational characteristics including HAI performance. In advance of the site visit, hospital leadership was provided with a list of desired key informants (executives, clinical leaders, infection prevention staff, and nurses) for interviews during the in-person site visit. Clinical leaders assisted with recruiting staff for interviews.

As previously described, the larger study sample included 18 hospitals that varied with respect to their CLABSI and CAUTI rates compared to the national average (i.e., better, average, or worse) based on Hospital Care Compare data [10]. Other hospital characteristics such as geographic region and association with an academic institution were also considered to increase variety in our study sample. In the larger study [11] and across all hospitals, we held in-person interviews with 471 key informants including hospital administrators, frontline staff (e.g., physicians and nurses), and leaders from departments such as infectious diseases, infection control, and epidemiology. This sub-analysis focused on the 10 higher-performing hospitals rated as better or no different than the national benchmark for CLABSIs and CAUTIs and included a total of 245 informants across all roles described above.

### 2.3. Data Collection

One-on-one and group interviews were conducted in person using a semi-structured interview guide that included questions about management practices surrounding their hospital’s efforts in HAI prevention. Topics covered in the interviews included the following: Goal Setting and Support; Strategic Alignment/Communication and Information Sharing; Systematic Education; Interprofessional Collaboration; Meaningful Use of Data; and Recognition for Success. The focus of this smaller study centered around when an occurrence of a CLABSI or CAUTI was reported (e.g., what was done and by whom). Study team members conducted interviews in staff breakrooms and hospital conference rooms. Interviewers included thirteen members of the research team who were MS-, ScD-, or PhD-trained health services and healthcare management researchers. Interviewees did not know their interviewer prior to their interview. All interviews were audio-recorded, transcribed verbatim, and de-identified.

### 2.4. Data Analysis

Interview transcripts were studied using deductive and inductive thematic analysis [12]. Three research team members who were MS- or PhD-trained health services researchers coded a small sample of transcripts using a preliminary coding dictionary developed from topics in the interview guide. Coders met at a minimum of weekly to discuss code definitions and make adjustments to the coding dictionary to ensure the consistency of coding. When the coding dictionary was finalized, the remaining transcripts were split among coders and codes were applied across all transcripts. Coders continued to meet as needed to resolve questions about the application of codes through collective consensus. This approach allowed for the consistent categorization of data based on general themes derived from the interview guide. The development of themes from codes was part of our iterative analytic process and occurred during our weekly coding and analysis meetings. HAI performance monitoring was one theme identified from the coded transcripts through our deductive analysis. Our inductive analysis involved the constant comparative method and enabled us to identify emergent themes around best practices for HAI performance monitoring and feedback. Comparison of themes across sites allowed us to characterize differences in management practices between sites that were higher- or lower-performing with respect to HAI prevention. The data presented in this study are focused on the perspectives of individuals at hospitals with average or better performance with respect to CLABSI and CAUTI rates compared to the national average. ATLAS.ti qualitative analysis software (ATLAS.ti Scientific Software Development GmbH, Berlin, Germany) was used to support the analysis of transcripts from the 10 higher-performing sites.

### 2.5. Ethical Considerations

The Institutional Review Board of our institution approved this study. Verbal informed consent was obtained from all subjects participating in the study.

## 3. Results

### 3.1. Hospital Characteristics

A majority of the interviewees who discussed management practices around HAI performance monitoring and feedback were from 10 hospitals that had been identified a priori as higher-performing: “better” with respect to HAI prevention or “average” in both CLABSI and CAUTI performance but had recently accelerated their infection prevention efforts. Interviewees at most of the lower-performing hospitals (i.e., the other eight hospital sites in our study) failed to provide examples of management practices related to HAI performance monitoring and feedback. The characteristics of the 10 higher-performing hospitals included in this sub-analysis are presented in Table 1.

### 3.2. Best Practices in HAI Monitoring and Feedback

Across hospitals, comments by interviewees at higher-performing sites revealed a common theme that robust practices were in place around HAI performance monitoring and feedback. Interviewees described the practices including specific management practice elements, ways of promoting safety culture, and the roles of those leading HAI performance monitoring and feedback. We discuss each management practice and associated subthemes in more detail in the subsections that follow.

#### 3.2.1. Management Practice Elements of HAI Performance Monitoring and Feedback

At each of these hospitals, interviewees described HAI performance monitoring and review processes that involved three management practice elements: (1) a timely review of the HAI, often in real time and during regularly scheduled meetings; (2) active awareness and engagement of hospital leadership; and (3) multidisciplinary participation in HAI reviews. One participant described the timely review of HAIs in their organization, which were initiated when infections were communicated to the ‪director of the medical ICU immediately upon detection: “In real time, every time they discover a CAUTI, CLABSI, whatever, they bring it to our attention then. And we discuss it weekly in our HAC [hospital-acquired condition] meeting”. In a different hospital, a leader of Quality and Safety highlighted the importance of leadership engagement in HAI reviews by explaining, “A debriefing happens with the clinicians in real time, but it’s a debriefing that gets shared or re-debriefed at all levels of the organization, all the way up to the senior management team. So, everybody has the same appreciation for where the processes fell down, and where they can be improved”. A description of the third management practice was provided by a leader in Infection Prevention who explained how HAI performance monitoring and feedback processes involved multidisciplinary participation in their organization, “There are event reviews for CLABSI and CAUTI. It used to be just infection prevention and the nurses. And now we have the attending, the respiratory therapy if they need to be there, the dialysis team. So, all those participants participate in that event review”. Additional representative quotations about these elements of HAI performance monitoring and feedback are presented in Table 2.

#### 3.2.2. Promoting Safety Culture in HAI Performance Monitoring and Feedback

Also salient across higher-performing hospitals was the promotion of safety culture in HAI performance monitoring and feedback which included stressing the importance of framing infection reviews as examinations of the failures of processes and procedures and not as ways to establish blame or punish staff. For example, an executive leader explained the focus on processes: “The nurse and the shift lead or assistant nurse manager or manager will present what they found in that 8 AM bed huddle, and that kind of makes everybody have that ownership of it. It’s not one person, it’s usually the process. Did we follow the process?” A medical director elaborated that the HAI performance monitoring and feedback process should not place blame on the individual, “You know, trying to make it less about whose fault is it and, you know, assigning blame, to: ‘Let’s fix the system. It is a system problem. Where can the system improve?’ Rather than pointing at an individual doctor or nurse who you know wasn’t following what they should’ve done. And how to improve different things like timeouts and those things”. Additional representative quotations describing these practices are presented in Table 3.

#### 3.2.3. Using HAI Performance Monitoring and Feedback to Identify Opportunities for Improvement

Finally, the goal of identifying opportunities for improvement when HAIs occurred and in preventing future infections was described by interviewees from multiple disciplines across these higher-performing hospitals. We found that the presentation of these opportunities for improvement could be led or driven by individuals with different roles. For instance, a manager of quality and patient safety explained a HAI review process where infection prevention took the lead: “So, if we had a CAUTI, we would actually, the IP [infection preventionist] would come and tell us [the opportunity for improvement committee] about what she found, and the investigation, and who worked on it, and what we could do to improve it.” A unit director of another hospital shared that their process for HAI review was driven by staff leadership at the unit level, “So, when one is identified on the unit, we will go back and talk with them, have a meeting with the attending and someone from infection prevention, members of their team who lead the unit. And then sometimes a staff nurse, but certainly staff leadership. And we go through a process that looks at … what was going on with the patient at the time, and did we have any opportunities there that were missed, pieces of the bundle basically, or anything unique to that case”. Quotations providing examples of these different approaches to leading HAI performance monitoring and feedback processes are presented in Table 4.

## 4. Discussion

Our study revealed important core elements of management practices for CLABSI and CAUTI performance monitoring and feedback. These core elements included timely and rigorous HAI reviews, strong leadership engagement, and regular involvement of multidisciplinary staff. These findings provide greater nuance to existing guidance surrounding broader HAI performance monitoring and feedback [13]. Our study findings also highlight the importance of identifying opportunities for improvement in infection prevention, regardless of which member of the healthcare team leads the infection review process. These results demonstrate that there are multiple approaches to HAI performance monitoring and feedback that are in use at higher-performing hospitals, and these may be contributing to their lower infection rates. What appears to be most important is that the critical elements of the performance monitoring and feedback process are carried out in a way that is embraced by both hospital leaders and frontline staff [14,15,16,17].

Of note, prior studies have acknowledged that fostering a safety culture is an important element of successful infection prevention programs [7,18]. In alignment with this evidence, we found that interviewees from higher-performing hospitals did not assign blame to staff when evaluating CLABSIs and CAUTIs. Emphasizing the importance of safety culture may be another way to support productive HAI performance monitoring and feedback processes by framing investigations as an opportunity to inform process improvements that can then prevent future infections.

During the COVID-19 pandemic, the National Steering Committee for Patient Safety released an urgent call to improve patient safety because of the worrisome rise in HAIs [19,20,21,22] emphasizing the need for additional efforts in infection prevention. As the pandemic has transitioned to an endemic phase and CLABSI and CAUTI rates seem to be improving [23,24,25], patient safety is still of paramount importance. Focusing on management practices specific to performance monitoring and feedback used in high-performing hospitals may provide a useful structure through which to investigate deficits in practice and to identify core elements of these processes that can be strengthened to achieve improvements in patient safety outcomes. Identifying opportunities for improvement in infection prevention using best management practices in HAI performance monitoring and feedback remains critical to creating and maintaining quality and safety in the healthcare environment.

Our study has several limitations. As this work was part of a larger project investigating multiple management strategies for CLABSI and CAUTI prevention, it is possible that a study focused specifically on management practices related to performance monitoring and feedback processes at lower-performing sites may reveal additional information that did not emerge in our study. While we utilized CLABSI and CAUTI performance ratings to select higher- and lower-performing hospitals, it is important to acknowledge that performance ratings are assigned at the hospital level while management practices often vary at the unit level, depending on standard operating procedures or protocols for a particular unit or service. The potential relationship between performance ratings and management practices is therefore complex, such that we cannot directly attribute infection prevention performance to the management practices reported by our study participants.

## 5. Conclusions

Participants in our study described management practices around conducting timely reviews of HAIs, ensuring hospital leadership’s engagement in the process, and securing the involvement of multidisciplinary members of the healthcare team, all of which may have contributed to their success in HAI prevention. Sharing the findings of these HAI reviews with hospital staff as learning opportunities can help foster a patient safety culture where investigating infections is never punitive and may help advance infection prevention efforts and, ideally, help hospitals achieve lower HAI rates.

## Figures and Tables

**Table 1:** Hospital characteristics of the 10 higher-performing sites selected for sub-analysis.

Site	CAUTI Performance ^1^	CLABSI Performance ^1^	Hospital Size ^2^	Academic Teaching Hospital	Region
1	Better	Average	Extra Large	Yes	Midwest
2	Average	Better	Medium	No	South
3	Average	Better	Large	Yes	Northeast
4	Average	Better	Medium	Yes	Northeast
5	Average	Average	Small	Yes	Midwest
6	Average	Average	Small	No	Midwest
7	Better	Average	Large	Yes	Midwest
8	Better	Better	Large	Yes	Northeast
9	Better	Better	Extra Large	Yes	South
10	Average	Better	Small	No	South

^1^ CAUTI and CLABSI performance data were acquired from the Centers for Medicare & Medicaid Services (CMS) Hospital Care Compare data. ^2^ Hospital size was defined by the number of beds: Small = less than 300 beds; Medium = 300 to 499 beds; Large = 500 to 899 beds; and Extra Large = 900 or more beds.

**Table 2:** Management practice elements of HAI performance monitoring and feedback.

Best Practices	Representative Quotes
Timely reviews	We get an email immediately, so we know as soon as infection prevention has confirmed it. It goes into our patient safety alert system. And then … the manager identifies hopefully somebody who was involved in that case, an RN, to review the infection.
	We learned that with CLABSI and CAUTI, you have to look at it every day. And nothing is assumed to just happen by chance.
Leadership engagement	We’ve always had the CLABSI event reviews … so there is more awareness and accountability. So, we get participation, and when it drifts away a little bit, we get a push from our executive sponsor to make sure that everyone is participating.
	We get an email sent to the leadership of that unit, as well as the hospital senior leadership. And, if it hasn’t included the intensivists, then we will send it on to the intensivists too so that way they are aware. It goes into our patient safety alert system.
Multidisciplinary participation	It’s multidisciplinary and that information is actually rolled out back to the front-line staff as well. So, if it happened on unit [name], the nursing director, the bedside nurse, hopefully the attending, and dialysis if they were involved, would all participate and do a drill down on what could we have done better to improve the outcome of this patient to prevent a CLABSI or a CAUTI.
	At huddle, we go over opportunities. We will offer discussions, you know. Sometimes people will interact or ask questions. … We also consult with our infection preventionist. You know, is there anything from your perspective that we missed, or we could have done better … ? Just to get the whole perspective.

**Table 3:** Promoting safety culture in HAI performance monitoring and feedback.

Best Practices	Representative Quotes
Focus on process	There’s usually a huddle on reporting about how that occurred, what we could have done different, what we could have improved on, was there anything at all? So, I think that they are very good about, on an individual basis, kind of recapping the things that we could have done differently, and the areas that we may have missed, and ways to improve.
	We have a weekly round-up here. Where all of nursing, and it’s a multidisciplinary meeting every Friday at 10 am, where we discuss let’s say, any hospital-acquired infections. We do a drill down. It gets presented to the entire team. And the teams actually present their fallouts with the help of the infection control department. That’s also a meeting where we have the opportunity to introduce new algorithms or introduce new practices. So, this round-up is a great forum for us.
Not assigning blame	Staff really, I feel, really want to do the right thing. They really do. I believe their hearts are really in the right place. So, with this [HAI review], I think it’s, you know, we do the no blame, because usually it’s sort of a few, it’s a period of time that we miss something, right?
	These event reviews are fairly time-consuming. Nobody wants to do it. I wouldn’t say they’re punitive, but they’re somewhat, it’s an opportunity to improve. It’s a no-blame environment. It’s just like, what did we do wrong?

**Table 4:** Role leading HAI performance monitoring and feedback.

Role	Representative Quotes
Infection Control	Between the manager and infection control, we each review the chart. What did we do right? What did we do wrong? Bring it to our attention, whether it was something as simple as you didn’t change an outside Foley, to you didn’t document good care, to whatever.
	We receive notification as soon as a CLABSI is identified from infection prevention. It gets sent to quality, it is sent to nursing, it is sent to physician leaders. And then our clinical nurse specialists do what they call a deep dive. So, they get in the patient’s chart, and they start looking at all sorts of things which includes was the line changed when it was supposed to be? Was there a documented need for the line? Why is the line still there? Have you done a good job documenting? And any gaps or errors in which he or she can see about the case. And then our quality committees or our safety committee will often go through those details to understand, you know, to learn from that. Because we still have CLABSIs.
Unit Leadership	As a unit, what our leadership did was at our morning safety huddles and evening safety huddles was they would review what were the risk factors that this patient had for developing infection, and what were some of the modifiable things that we could have done as nursing to prevent that.
	We are having an open discussion on what was done, what can we do better. And when I was the quality rep [resentative], I took that information back and educated my staff. I can’t answer for every unit or every person, but I know what I did. And I mean, we went two years without a CLABSI. And it was just holding people accountable.

## Data Availability

Due to participant privacy concerns, the data presented in this study are not publicly available. The data may be requested from the corresponding author.

## Public Involvement Statement

There was no public involvement in any aspect of this research.

## Guidelines and Standards Statement

This manuscript was drafted against the Consolidated Criteria for Reporting Qualitative Research (https://www.equator-network.org/, accessed on 2 February 2024).
